# The coronavirus disease 2019 (COVID-19) delta wave: Refocusing effort and remaining resilient in the face of evolving infection prevention and antimicrobial stewardship challenges

**DOI:** 10.1017/ash.2021.211

**Published:** 2021-11-18

**Authors:** Michael P. Stevens, Jacob W. Pierce, Rachel Pryor, Michelle E. Doll, Gonzalo M. Bearman

**Affiliations:** 1 Division of Infectious Diseases, Department of Internal Medicine, Virginia Commonwealth University School of Medicine, Richmond, Virginia; 2 Virginia Commonwealth University Health System, Richmond, Virginia

**Keywords:** COVID-19, infection prevention, antimicrobial stewardship, burnout

## Abstract

Challenges for infection prevention and antimicrobial stewardship programs have arisen with the fourth wave of the coronavirus disease 2019 (COVID-19) pandemic, fueled by the delta variant. These challenges include breakthrough infections in vaccinated individuals, decisions to re-escalate infection prevention measures, critical medication shortages, and provider burnout. Various strategies are needed to meet these challenges.

The United States is currently in the midst of an escalating “fourth wave” of coronavirus disease 2019 (COVID-19) activity driven by the highly infectious delta variant. This surge follows a brief period of declining COVID-19 activity in the United States associated with widespread vaccination that led to the de-escalation of numerous community infection prevention measures.

Healthcare systems across the United States are now facing increasing COVID-19 admission rates as well as employee infections. Major challenges are facing infection prevention and antimicrobial stewardship programs: vaccine breakthrough infections, supply chain issues with COVID-19–focused therapeutics, and a profoundly fatigued work force.

## Vaccines and novel therapeutics

**Fig. 1.**
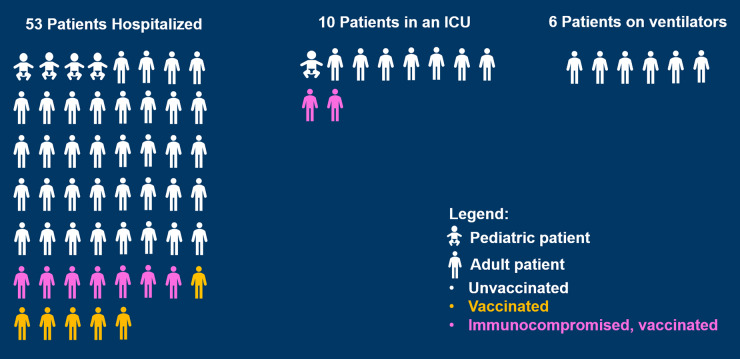
Contextualizing breakthrough infections at an academic medical center (data for August 17, 2021).

Although 3 COVID-19 vaccines are available in the United States and they have proven highly effective at preventing serious infection, hospitalization, and death, breakthrough infections are now well documented and have undermined trust in vaccination. Importantly, even when breakthrough infections occur, they tend to be mild and rarely result in hospitalizations.^1^ Although most infections in the United States occur among the unvaccinated, some fully vaccinated people are being hospitalized with symptomatic disease. Providing a meaningful context for the benefits of vaccination (even for admitted patients) is critical. Our medical center created a graphic to show the benefit of vaccination even in people who are sick enough to be hospitalized despite vaccination (Fig. 1).

Healthcare systems must adapt rapidly and nimbly to the evolving COVID-19 vaccine science regarding third doses of mRNA vaccines (ie, boosters). Health systems will need to rapidly operationalize the logistics of offering this to employees. Many health systems will need to do this simultaneously with influenza vaccination programs. For health systems that have mandated COVID-19 vaccination, it is not clear how recommendations for a “booster” dose of vaccine will best be incorporated into an existing COVID-19 vaccine mandate.

After enjoying a brief period of reliable novel therapeutic medication access in the United States, health systems are once again facing critical shortages. Tocilizumab, an IL-6 inhibitor being used for its anti-inflammatory properties, has found a niche in treating severely ill inpatients with COVID-19, particularly those with rapid respiratory decline. Many health systems now have limited or no access to this medication.^3^ Similarly, monoclonal antibodies have found an important role in both the treatment of and prophylaxis for severe acute respiratory coronavirus virus 2 (SARS-CoV-2) infection. If the available monoclonal antibodies were deployed as broadly as their FDA emergency use authorization would allow, the supply chain for these agents would be potentially overwhelmed. Health systems that are already facing strain from surging COVID-19 activity must build and expand their infrastructures to be able to deliver therapy systematically and equitably. Acquiring the financial capital to supply the human resources necessary to expand this infrastructure is particularly problematic; healthcare systems continue to log record financial losses and are contending with significant staffing shortages.

## Breakthrough infections and fluctuating infection prevention strategies

Breakthrough infections have also called into question using vaccination status in various infectious prevention risk-stratification strategies. Reports from Israel suggest increasing rates of severe COVID-19 disease among older vaccinated individuals with comorbidities who had received second vaccination >5 months previously.^1,2^ Many healthcare systems deployed entry screening as well as broad preprocedural testing earlier in the pandemic. With widespread vaccination and declining community COVID-19 activity, some hospitals have been using vaccination status to limit testing in asymptomatic patients in both settings. Entry and preprocedural testing for asymptomatic patients is costly and can lead to delays in care. Additionally, vaccinated health-system staff who are utilizing personal protective equipment correctly would have very low risk for acquiring infection from an asymptomatic patient with SARS-CoV-2 infection. Although staff risk may be relatively low, health systems do have to consider patient to patient spread, especially systems that utilize multiple occupancy rooms. The most optimal hospital entry and preprocedural testing strategies are still unclear, especially in the setting of new SARS-CoV-2 variants and the current pandemic surge.

Healthcare systems must also aggressively readdress the importance of nonpharmaceutical interventions for limiting transmission in the healthcare system. Where local caseloads are increasing, healthcare systems will need to adjust local restrictions to limit previously identified risk factors for infection. Such measures could include occupancy restrictions for rooms, visitation, or travel policy restrictions, and encouraging virtual meetings where possible. More advanced measures, such as monitors to ensure appropriate donning/doffing of personal protective equipment, could be implemented depending on local staffing and COVID-19 caseloads.^4^ All healthcare systems should also stress the importance of healthcare workers staying out of the clinical environment if they feel ill.

## Burnout and workforce fatigue

Importantly, re-escalating infection prevention measures puts both financial and psychological strain on healthcare systems and employees. Another critical concern is fatigue among healthcare workers, which may lead to a reduced focus on maintaining compliance with optimal infection prevention best practices. It may also lead frontline staff to leave their current jobs, which imposes stress on organizations during the current COVID-19 wave.

Organizational-level interventions that may help to mitigate burnout include mindfulness training, stress management seminars, small group discussions, and formal duty-hour reductions and/or workload reductions.^5^ A practical reality, however, is that profoundly stressed healthcare systems currently need critical staff and space to treat patients, as well as the money to recruit and retain personnel and to create infrastructure. An important challenge facing infection prevention and antimicrobial stewardship program leaders is the issue of leading teams when staff are exhausted. Although no evidence-based guidelines exist for best mitigating the risk of burnout in this setting several points are notable. On an individual level, leaders should be aware of personal and team resilience to set clear expectations with limits based on what is deemed urgent versus what is important. Priorities should be adjusted based on feedback, with major emphasis on limiting unnecessary tasks that erode valuable time.^6^ A primary leadership goal is to balance compassion and crisis containment by recognizing people for who they are as well as what they do.^6^ A recent report suggests that teams are generally most effective when work is purposeful, personally integrated, and not just focused on efficiency.^7^ Leaders must recognize and mitigate burnout in both their teams and themselves.

In conclusion, the fourth wave of COVID-19 infection in the United States, fueled by the delta variant, is rapidly evolving. Vaccination may play an important role in thwarting escalating cases, but it is not clear whether adoption will be widespread enough or will occur soon enough to have a significant impact on this current wave. Healthcare systems must continue to encourage initial vaccination, and they will need to adopt new policies concerning potential booster vaccinations. Healthcare systems face major challenges in both accessing and distributing novel COVID-19–focused therapeutics. The adoption of a wide array of preventive strategies is critical for healthcare systems to reduce the spread of SARS-CoV-2, but these strategies may be associated with significant organizational financial and psychological stress. Although healthcare workers have demonstrated resilience despite myriad challenges, healthcare systems should acknowledge the significant risk of burnout among both frontline personnel and leaders.
